# How Mobile App Design Impacts User Responses to Mixed Self-Tracking Outcomes: Randomized Online Experiment to Explore the Role of Spatial Distance for Hedonic Editing

**DOI:** 10.2196/mhealth.9055

**Published:** 2018-04-11

**Authors:** Monika Imschloss, Jana Lorenz

**Affiliations:** ^1^ Department of Retailing and Customer Management University of Cologne Cologne Germany

**Keywords:** mobile apps, self-tracking, user interaction design, goal setting

## Abstract

**Background:**

Goal setting is among the most common behavioral change techniques employed in contemporary self-tracking apps. For these techniques to be effective, it is relevant to understand how the visual presentation of goal-related outcomes employed in the app design affects users’ responses to their self-tracking outcomes.

**Objective:**

This study examined whether a spatially close (vs distant) presentation of mixed positive and negative self-tracking outcomes from multiple domains (ie, activity, diet) on a digital device’s screen can provide users the opportunity to hedonically edit their self-tracking outcome profile (ie, to view their mixed self-tracking outcomes in the most positive light). Further, this study examined how the opportunity to hedonically edit one’s self-tracking outcome profile relates to users’ future health behavior intentions.

**Methods:**

To assess users’ responses to a spatially close (vs distant) presentation of a mixed-gain (vs mixed-loss) self-tracking outcome profile, a randomized 2×2 between-subjects online experiment with a final sample of 397 participants (mean age 27.4, SD 7.2 years; 71.5%, 284/397 female) was conducted in Germany. The experiment started with a cover story about a fictitious self-tracking app. Thereafter, participants saw one of four manipulated self-tracking outcome profiles. Variables of interest measured were health behavior intentions, compensatory health beliefs, health motivation, and recall of the outcome profile. We analyzed data using chi-square tests (SPSS version 23) and moderated mediation analyses with the PROCESS macro 2.16.1.

**Results:**

Spatial distance facilitated hedonic editing, which was indicated by systematic memory biases in users’ recall of positive and negative self-tracking outcomes. In the case of a mixed-gain outcome profile, a spatially close (vs distant) presentation tended to increase the underestimation of the negative outcome (*P*=.06). In the case of a mixed-loss outcome profile, a spatially distant (vs close) presentation facilitated the exact recognition of the positive outcome (*P*=.04). When the presentation of self-tracking outcomes provided the opportunity for hedonic editing, users with a low (vs high) health motivation produced compensatory health beliefs, which led to lower health behavior intentions (index of moderated mediation=0.0352, 95% CI 0.0011-0.0923).

**Conclusions:**

When spatial distance between the presentations of mixed self-tracking outcomes provided the opportunity to hedonically edit one’s self-tracking outcome profile, users recalled their self-tracking outcomes in a more positive light. Especially for users with lower health motivation, the opportunity to hedonically edit one’s mixed self-tracking outcome profile led to reduced health behavior intentions. To prevent the occurrence of hedonic editing in users’ responses to visually presented self-tracking outcome profiles, further research is necessary to determine the ideal distance that should be employed in the app design for the presentation of mixed self-tracking outcomes on a digital device’s screen.

## Introduction

### Background

Given the growing spread of wearable technological devices and mobile phones alongside with mobile apps, consumers nowadays increasingly practice digital forms of self-tracking [1,2], which is the systematic recording of “information about one’s diet, health, or activities...so as to discover behavioral patterns that may then be adjusted to help improve one’s physical or mental well-being” [3]. The rise of apps encouraging the self-tracking of physical activity and diet [4], such as the popular “Lose It!” or “MyFitnessPal” apps, enable consumers to easily keep a digital record of calorie burn and consumption. Commonly, these apps allow users to set a daily caloric goal and to monitor the amount of calories burned compared to the amount of calories consumed. Through this, these apps aim to spur users to live a healthy life and to facilitate the pursuit of personal health goals such as weight loss or weight maintenance [5,6]. Also, researchers advocate that apps might be promising tools that can contribute to the promotion and improvement of people’s overall health [7,8].

So far, existing studies have examined the effectiveness of health-related mobile phone interventions and technology-enhanced interventions in general [9-15] or have assessed the prevalence and effectiveness of behavioral change techniques employed in the app design in particular [16,17]. The use of a tracking app in general has been associated with increased behavioral intentions [18], and goal setting is one of the behavioral change techniques commonly employed in self-tracking apps [7,8,19], yet little is known about how the presentation of goal-related outcomes from different domains (eg, activity, diet) employed in a self-tracking app impacts users’ self-reflection of the collected data.

**Figure 1:**
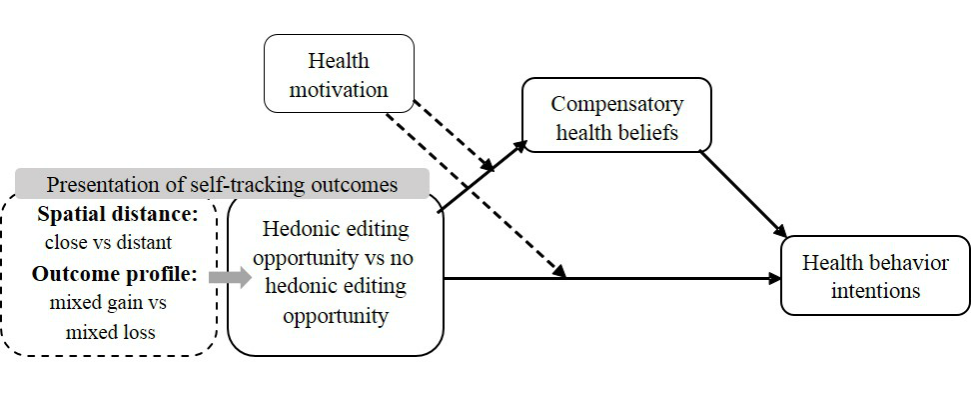
Proposed model of how hedonic editing opportunity as a function of spatial distance and self-tracking outcome profile influences users’ health behavior intentions. The self-tracking outcome profile is a mixed gain if there is a positive outcome in one domain and a smaller negative outcome in the other domain. Conversely, in a mixed-loss outcome profile, there is a large negative outcome in one domain and a smaller positive outcome in the other domain.

This lack of research is surprising, considering that users’ reflections of their self-tracking outcomes most likely will determine their health-related intentions and actions [20]. Accordingly, it is relevant to understand how features of outcome presentation, such as the spatial distance between goal-related outcomes on a digital device’s screen as it is employed in the app design, can affect how users respond to their self-tracking outcomes. In view of the lack of health behavior theory integration in the development of health apps in general [21,22], this research transfers mental accounting principles of hedonic editing [23], originally from the field of economics, to the context of self-tracking. Based on this theoretical approach, the objective of this research is to examine whether, depending on the overall outcome profile, a spatially close compared to a distant presentation of mixed positive and negative self-tracking outcomes from the domains of physical activity and diet can provide users the opportunity to hedonically edit their self-tracking outcome profile (ie, to view their mixed self-tracking outcomes in the most positive light). This study further provides a motivated cognitive justification account to examine how the opportunity to hedonically edit one’s self-tracking outcome profile relates to users’ future health behavior intentions. For an overview of the study’s framework, see Figure 1.

### Theory and Hypotheses Development

#### Mental Accounting and Principles of Hedonic Editing in the Context of Self-Tracking

First, to apply principles of mental accounting—originally used to describe how consumers mentally code, categorize, and evaluate multiple economic outcomes [23,24]—to the context of self-tracking, we argue that when physical activity and dietary self-tracking outcomes are measured along the same dimension (ie, calories), this allows for the combination of both outcomes within a mental account of health. This argument is based on the reason that outcomes from both domains influence one’s total energy balance, which relates to overall health.

Second, to describe how consumers mentally combine multiple outcomes within a single account, Thaler [23] draws on the shape of prospect theory’s value function [25] and proposes that consumers perceive their outcomes relative to a reference point either as gains or losses. Accordingly, in a self-tracking context, setting caloric goals in the domain of physical activity as well as diet can serve as reference outcomes relative to which mobile self-tracking app users classify each of their self-tracking outcomes either as a gain (positive outcome; eg, burning more calories from physical activity than the caloric goal) or a loss (negative outcome; eg, burning less calories from physical activity than the caloric goal). We propose that a mixed outcome profile—such as a positive outcome in one domain and a negative outcome in the other domain—is inherently ambiguous and thus leaves some scope for users’ subjective evaluation.

Third, to explain how consumers evaluate combinations of positive and negative outcomes, Thaler [23,24] has derived principles of hedonic editing that are based on the shape of the value function. According to Thaler’s [23] principles of hedonic editing, consumers prefer to evaluate mixed outcomes either jointly (integration) or separately (segregation) to maximize their happiness. As such, principles of hedonic editing predict that when the combined outcome is a mixed gain, which is the case if there is a positive outcome in one domain and a smaller negative outcome in the other domain, users strive to integrate the negative with the positive outcome [23]. This is because the integration of the small negative outcome into the positive outcome allows one to cancel out the pain from the negative outcome and reduces the recognition of the loss [26]. Conversely, if the combined outcome is a mixed loss, which is the case if there is a large negative outcome in one domain and a smaller positive outcome in the other domain, hedonic editing principles suggest that users strive to segregate the positive from the negative outcome [23]. This is because the small positive outcome becomes a “silver lining” in the face of the large negative outcome and segregation versus integration allows consumers to appreciate the positive outcome even when the combined result is negative [23,26]. Taken together, principles of hedonic editing suggest that when self-tracking app users experience an overall mixed-gain self-tracking outcome profile, they would look for ways to integrate the small negative outcome. Conversely, when users experience an overall mixed-loss outcome profile, they would look for ways to segregate the small positive outcome.

#### Spatial Distance and Hedonic Editing of Self-Tracking Outcomes

Regarding the influence of spatial distance on the use of hedonic editing strategies, we draw on previous research, which suggests that temporal distance between the occurrence of a positive and negative outcome facilitates hedonic editing [26,27]. As such, temporal separation facilitates the recognition of the positive event as a distinct occurrence, whereas temporal closeness provides the opportunity to integrate and cancel out the negative occurrence [26]. Consistent with this reasoning, we propose that spatial distance in the presentation of multiple self-tracking outcomes can facilitate hedonic editing. In particular, we assume that in the case of a mixed-gain outcome, a close versus distant presentation of self-tracking outcomes will help integrate the small negative outcome into the larger positive outcome and provide the opportunity to hedonically edit one’s self-tracking outcomes. Conversely, in the case of a mixed-loss outcome profile, a distant versus close presentation of self-tracking outcomes will help segregate the small positive outcome from the larger negative outcome and provide the opportunity for hedonic editing.

Following the reasoning of Cowley [26], we propose that hedonic editing is indicated by users’ allocation of attention and the resulting accuracy when recalling the caloric values of the small negative outcome or the small positive outcome, respectively. As such, in case of a mixed-gain outcome profile, a close compared to a distant presentation of self-tracking outcomes should facilitate hedonic editing (ie, the integration of the small negative outcome into the larger positive outcome), which would be reflected by an underestimation of the small negative outcome. Conversely, in the case of a mixed-loss outcome profile, a distant compared to a close presentation of self-tracking outcomes should segregate the small positive outcome from the larger negative outcome and hedonic editing would be indicated by a more accurate recall of the small positive outcome.

Therefore, our first hypothesis is that, in the case of a mixed-gain outcome profile, a close (vs distant) presentation of self-tracking outcomes will lead to an underestimation (vs accurate estimation or overestimation) of the recalled small negative outcome and that, in the case of a mixed-loss outcome profile, a distant (vs close) presentation of self-tracking outcomes will lead to a more accurate (vs overestimation or underestimation) memory of the small positive outcome.

#### Hedonic Editing Opportunity and User Responses to Self-Tracking Outcomes

Because mental accounts are considered to function as self-regulatory mechanisms [28], the opportunity to hedonically edit one’s mixed self-tracking outcomes might affect users’ responses to their self-tracking outcomes such as their health behavior intentions (eg, intending to do more sports or to eat healthier in the future). We propose that when the opportunity to hedonically edit mixed self-tracking outcomes arises (vs not), users—particularly users who have lower health motivation—will respond to this opportunity and reflect their outcomes in the best possible light, which will result in lower health behavior intentions.

We suggest that this effect might be explained by a motivated cognitive justification mechanism. We argue that particularly users with a low as opposed to a high health motivation may—probably unconsciously—justify using the opportunity to hedonically edit their self-tracking outcomes, which allows them to have lower health behavior intentions in response to their tracking results. One way to cognitively justify reduced health behavior intentions in response to the hedonic editing opportunity is the formation of compensatory health beliefs, which are “beliefs that the negative effects of an unhealthy behavior can be compensated for, or ‘neutralized,’ by engaging in a healthy behavior” ([29], p 607) and thus enable users “to justify unhealthy behavior choices” ([29], p 608). Therefore, we propose that users with a lower health motivation will have reduced health behavior intentions when the presentation of the outcome profile provides the opportunity for hedonic editing and that this effect occurs due to the activation of compensatory health beliefs. Formally, we hypothesize that users’ levels of health motivation will moderate the direct effect of hedonic editing opportunity on health behavior intentions as well as the indirect effect of hedonic editing opportunity on health behavior intentions through compensatory health beliefs.

## Methods

### Aims of the Study

This study aimed to establish that spatial distance in the presentation of self-tracking outcomes facilitates hedonic editing and examined whether a close compared to a distant presentation of self-tracking outcomes in the case of a mixed-gain or a mixed-loss outcome profile biased users’ memory of self-tracking outcomes as proposed by hedonic editing principles. Further, this study examined how the opportunity to hedonically edit one’s outcome profile affected users’ health behavior intentions. To investigate our hypotheses, we conducted a 2 (spatial distance: close vs distant) × 2 (outcome profile: mixed gain vs mixed loss) between-subjects online experiment.

### Recruitment

The experiment was designed in the German language using SoSci Survey, a software package for conducting online surveys. We tested the technical functionality of the electronic questionnaire and the correctness of electronic data recording as well as the transmission of collected data before making the online experiment public. A convenience sample was used because the invitation link to the open online survey was distributed in various groups of social media networks and shared on two survey websites. Consequences of this procedure for the survey population with respect to the sex (ie, more females) and age (ie, relatively young) were expected. We recruited participants from December 2016 to January 2017. The study was approved by the Head of the Department of Retailing and Customer Management, University of Cologne, Germany, to confirm compliance with ethical standards. As such, before beginning of the survey, participants were informed on the entry page about the general topic, purpose, and procedure of the study, and that their participation was voluntary and the data would be treated anonymously. Participants had to give their consent by clicking on the button “I agree” to continue with the study. No incentives were offered.

### Design and Procedure

The study employed a 2 (spatial distance: close vs distant) × 2 (outcome profile: mixed gain vs mixed loss) between-subjects design and questionnaires with the respective experimental condition were randomly displayed by the survey software. The cover story explained that the study’s aim was to optimize the design of a yet-unreleased self-tracking app that measures health-related information so that users can easily monitor their activity, diet, and overall energy balance. The instruction sheet stated that the study sought to find out how comprehensible the app layout was. We briefed participants about the integral elements of the fictitious self-tracking app and explained that they will see an illustration of self-tracking results as they were obtained at the end of a certain day, containing information about activity (calories burned from physical exercise), diet (calories consumed from food and drinks), and overall energy balance (difference between calorie burning and consumption). We told participants that the app sets daily caloric goals based on individual needs for the domain of activity (minimum amount of calories that should be burned from activity during the day) and diet (maximum amount of calories that should be consumed during the day). These goals should be achieved or surpassed to obtain an overall even energy balance, where calorie consumption does not exceed calorie burning. Thereafter, participants saw a fictive self-tracking outcome profile including the display of goal achievement in the domains of activity, diet, and overall energy balance. We asked participants to imagine that they themselves had obtained the depicted self-tracking outcomes at the end of a certain day.

**Figure 2:**
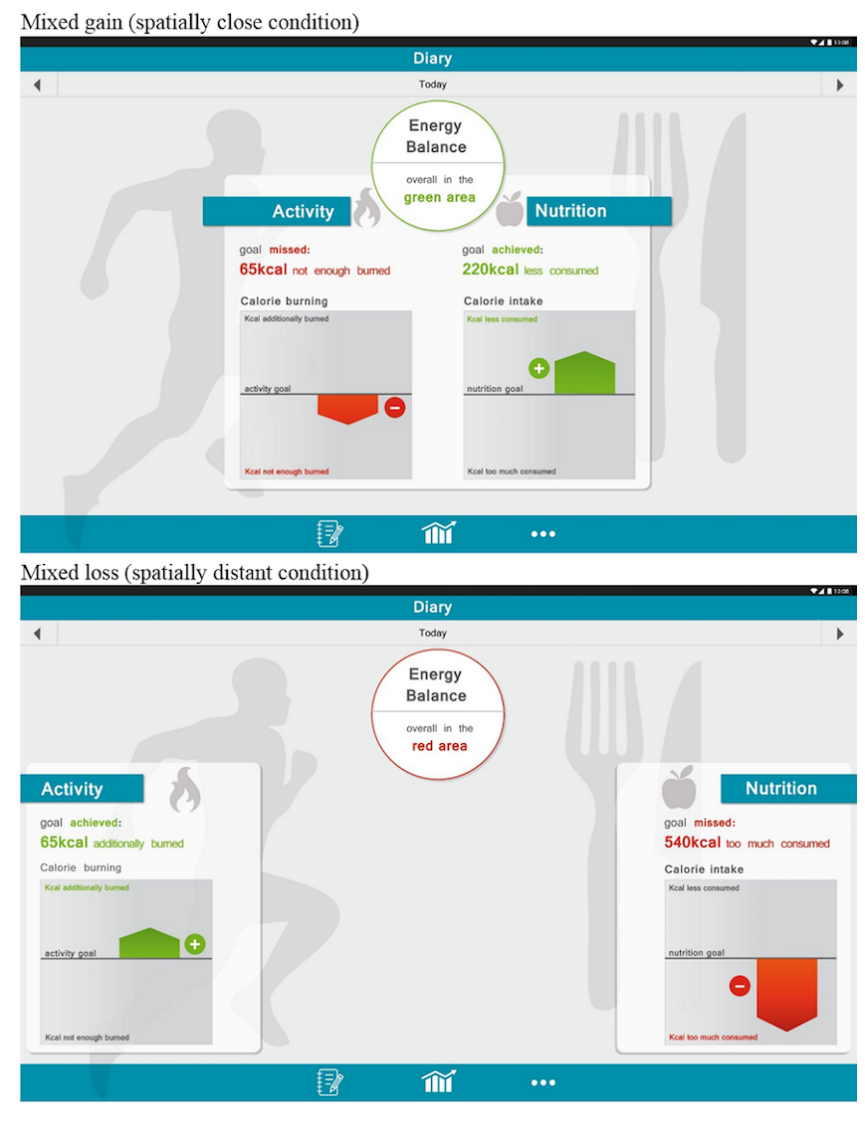
Illustration of mixed-gain and mixed-loss self-tracking stimuli.

We presented participants either a mixed-gain or mixed-loss self-tracking outcome profile with the outcomes in the domains of activity and diet being either spatially close to or distant from one another. The four different self-tracking outputs were designed with the graphic program Adobe InDesign CS6 (see Figure 2).

In the mixed-gain condition, the self-tracking results showed participants that they had underscored their caloric activity goal by 65 kcal and achieved their caloric diet goal by consuming 220 kcal less than the target amount of calories, hence having an overall positive energy balance. In the mixed-loss condition, the self-tracking results showed participants that they had exceeded their caloric activity goal by 65 kcal, but that they had failed to achieve their caloric dietary goal by consuming 540 kcal too much, hence having an overall negative energy balance. The exact goal value was not indicated to prevent participants from comparing the fictitious goal with their own needs or their own set goals. The outcomes of activity and nutrition were highlighted graphically through bar graphs and marked with plus or minus signs. The colors green and red were used for the plus and minus signs, respectively, and were consistently used in the output design to enable fast recognition of gains or losses. Spatial distance between self-tracking outcomes from the domain of activity and diet in the close condition was set to 94 pixels and in the distant condition to 667 pixels. Further, the output included information about the overall energy balance to prompt a mental connection between activity and nutrition.

### Measures

All measures took place immediately after participants had seen the self-tracking outcome profile. The questionnaire assessed participants’ health behavior intentions by asking how likely it was that they had activity-related and diet-related behavioral intentions in response to their self-tracking outcomes (four items on a 7-point scale anchored visually at 1=very unlikely and 7=very likely): “I want to be more active in the near future,” “I want to do more sports in the near future,” “I want to eat healthier in the near future,” and “I want to eat less high-caloric in the near future” (α=.70). Similar to previous work [29], participants’ compensatory health beliefs were measured by five items (on a 7-point scale anchored visually at 1=don’t agree at all and 7=totally agree): “If I eat less or more healthily, it is not necessary to do a lot of sports,” “It is okay to eat an unhealthy snack after having had a hard workout,” “It is okay to eat more during lunch and dinner if one has skipped breakfast in the morning,” “Skipping exercising in one week can be compensated for by exercising twice the next week,” and “Eating dessert is okay if one restrains eating during the main dish” (α=.68). To assess participants’ level of health motivation, we asked about the importance of activity and diet in their life (on a 7-point scale anchored visually at 1=don’t agree at all and 7=totally agree): “Doing sports is an important element in my life,” “I can easily live without doing sports” (reverse coded), “A healthy diet is important to me,” and “I eat unhealthy fast food very often” (reverse coded) (last item was excluded to increase α to .69). To test for a bias of participants’ attention focus and a consequential hedonic editing memory bias, participants were asked to recall the absolute caloric deviation from the activity as well as diet goal (open answer format). The questionnaire contained further measures on participants’ feelings and perceptions of the app as well as personal and demographic information (for details on measures, see Multimedia Appendix 1).

### Statistical Analysis

We analyzed our data using the statistical package of SPSS version 23 (IBM Corp, Armonk, NY, USA). All effects are reported as significant at *P*<.05. First, to test the first hypothesis, we analyzed whether spatial distance affected participants’ accuracy of recalled absolute deviations (open answers in kcal) from the determined goal in the domain of activity in line with hedonic editing principles. In the mixed-gain conditions, users had a small negative outcome in the domain of activity (65 kcal less burned than the set goal) and we examined whether a close compared to a distant presentation of self-tracking outcomes resulted in an underestimation (vs accurate estimation or overestimation) of the recalled small negative outcome. For this analysis, we binary coded the recalled kcal scores into whether they underestimated the caloric deviation of the small loss (<65 kcal) or whether they exactly matched or overestimated the caloric deviation (≥65 kcal). In the mixed-loss conditions, users had a small positive outcome in the domain of activity (65 kcal more burned than the set goal) and we tested whether a distant compared to a close presentation of self-tracking outcomes resulted in a more accurate (vs overestimation or underestimation) recall of the small positive outcome. For this analysis, we binary coded the recalled kcal scores into whether they exactly matched the caloric deviation (=65 kcal) or whether they either underestimated or overestimated the caloric deviation (<65 kcal or >65 kcal). For the mixed-gain and the mixed-loss conditions, a Pearson chi-square test with spatial distance and the according binary-coded response variable was conducted to examine whether users’ accuracy of recalled self-tracking outcomes changed as an effect of spatial distance in line with hedonic editing principles. We report the asymptotic two-sided significance of this test.

Second, to test our second hypothesis, we analyzed whether users’ levels of health motivation moderated the direct effect of self-tracking outcome presentation that either does or does not provide the opportunity for hedonic editing on health behavior intentions as well as the indirect effect of self-tracking outcome presentation on health behavior intentions through compensatory health beliefs. To simultaneously test both the moderator and mediator, we used the PROCESS macro version 2.16.1 for SPSS, which is suited for conducting a moderated mediation analysis (moderated mediation; model 8 [30]). We used bias *-* corrected bootstrap confidence intervals based on 10,000 resamples and the significance of the indirect effect was based on a 95% confidence interval. If the range of the upper and lower level confidence intervals does not include zero, the analysis indicates significance. The independent variable was coded 1 when the outcome profile provided the opportunity to hedonically edit one’s self-tracking outcomes (ie, mixed gain/close presentation and mixed loss/distant presentation) and was coded 0 when no hedonic editing opportunity was given (ie, mixed gain/distant presentation and mixed loss/close presentation). We set users’ level of health motivation as the moderator and compensatory health beliefs as the mediator. Users’ health behavior intentions served as the dependent variable.

## Results

### Sample

A total of 584 participants completed the online experiment. Manipulation checks served to exclude 187 participants who indicated they remembered a wrong outcome profile as well as participants who indicated they remembered an extremely high or low deviation from the determined caloric goals in the domain of activity (kcal values ≤10 and ≥450) or diet (kcal values ≤10 and ≥1000) in the free recall question on their goal deviation (kcal value). The final sample consisted of 397 participants with cell sizes ranging between n=84 and n=113. The sample consisted of 71.5% (284/397) female participants and the mean age was 27.4 (SD 7.2) years. In terms of the highest educational level attained, of the 397 participants, 2 (0.5%) had a lower secondary school leaving certificate, 33 (8.3%) had an intermediate or general secondary school leaving certificate, 154 (38.8%) had a general or subject-linked higher education entrance qualification, 133 (33.5%) had a bachelor’s degree, 73 (18.4%) had a master’s degree or a diploma, and 2 (0.5%) had a doctoral degree. We used participants’ voluntary information about body weight (in kg) as well as body size (in cm) to calculate the body mass index (BMI). For the 354 participants who voluntarily provided information, the mean BMI was 23.7 (SD 3.9) kg/m^2^.

**Figure 3:**
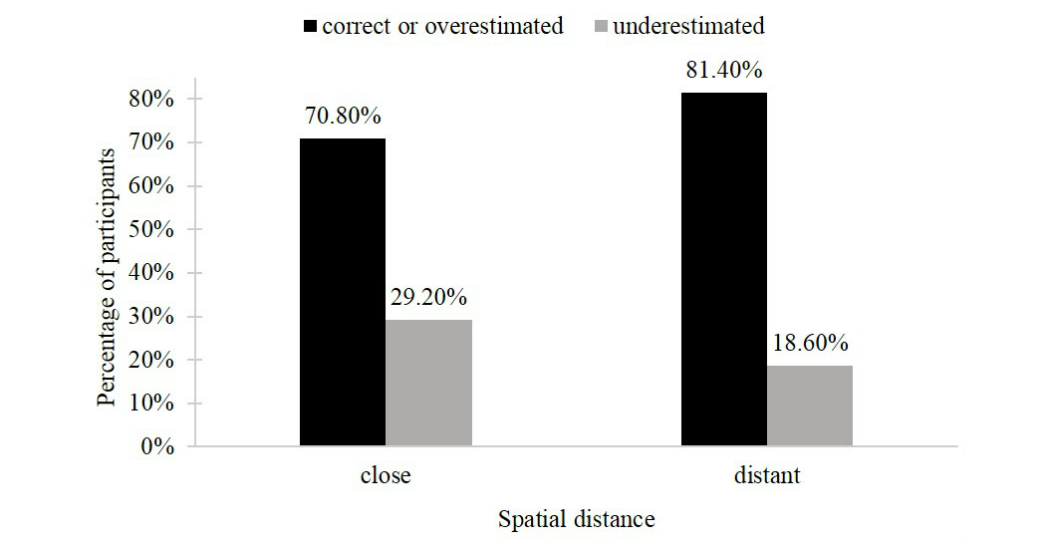
Recall of the small loss in the mixed-gain outcome conditions.

**Figure 4:**
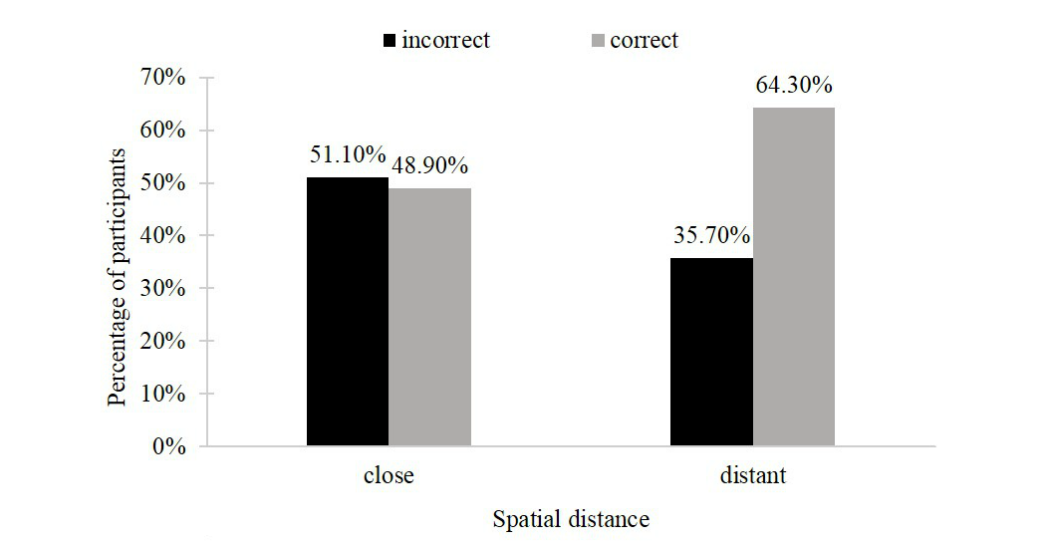
Recall of the small gain in the mixed-loss outcome condition.

### Hedonic Editing of Self-Tracking Outcomes

The first hypothesis assumed that for a mixed-gain outcome profile, a close rather than a distant presentation of self-tracking outcomes would lead to an underestimation versus an accurate estimation or overestimation of the recalled small negative outcome. The chi-square test was marginally significant and showed that when mixed-gain outcomes were presented close to (vs distant from) one another, a higher proportion of participants underestimated the small loss (31/106, 29.2% vs 21/113, 18.6%) and a lower proportion of participants exactly recalled or overestimated the small loss (75/106, 70.8% vs 92/113, 81.4%; χ^2^_1_=3.4, *P*=.06). The second part of the first hypothesis proposed that for a mixed-loss outcome profile, a distant rather than a close presentation of self-tracking outcomes would lead to an accurate estimate versus an overestimation or underestimation of the recalled small positive outcome. The chi-square test revealed a significant effect of spatial distance on accuracy of recalled kcal scores. The analysis indicated that in the distant (vs the close) mixed-loss condition 64.3% (54/84; vs 46/94, 48.9%) of participants recalled the small gain correctly and that 35.7% (30/84; vs 48/94, 51.1%) of participants recalled the small gain incorrectly (χ^2^_1_=4.2, *P*=.04). Figures 3 and 4 illustrate the results and display the percentage of participants underestimating the small loss in the mixed-gain condition and the percentage of participants correctly recognizing the small gain in the mixed-loss outcome conditions, respectively.

### Effect of Hedonic Editing Opportunity on Health Behavior Intentions

To test the second hypothesis of how the opportunity to hedonically edit one’s self-tracking outcome affects participants’ health behavior intentions, we examined the mediating role of compensatory health beliefs and the moderating role of health motivation (moderated mediation analysis, Table 1).

**Figure 5:**
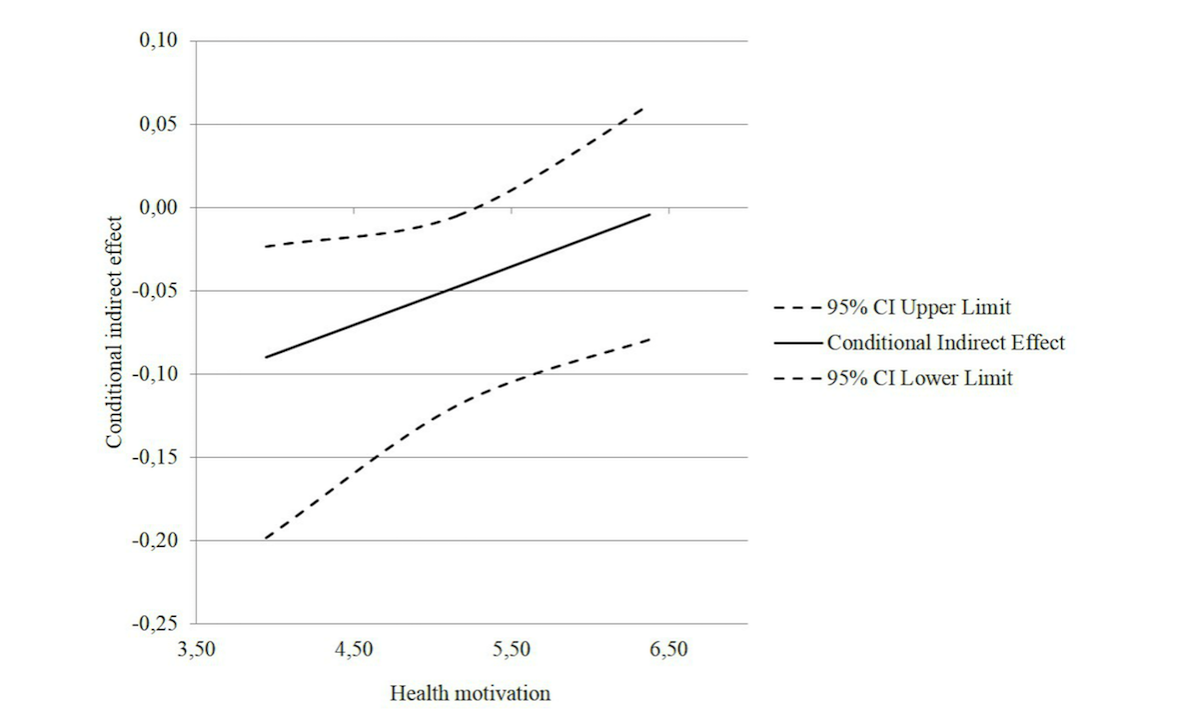
Conditional indirect effect of hedonic editing opportunity on health behavior intentions at values of the moderator health motivation through compensatory health beliefs.

The analysis revealed that health motivation was a marginally significant moderator of the association between hedonic editing opportunity and compensatory health beliefs (estimate of interaction=–0.17; *P*=.07), but not of the direct effect of the hedonic editing opportunity on health behavior intentions (estimate of interaction=0.02; *P=*.83). Further, the results showed that compensatory health beliefs were negatively related to health behavior intentions (*b*=–.21; *P*<.001). Results indicated that the effect of hedonic editing opportunity on health behavior intentions was moderated by health motivation and mediated by compensatory health beliefs (index of moderated mediation=0.0352, 95% CI 0.0011-0.0923). Results confirmed that especially users with a lower level of health motivation produce compensatory health beliefs in response to the hedonic editing opportunity, which decreased health behavior intentions. As such, in support of our second hypothesis, the conditional indirect effect of hedonic editing opportunity on health behavior intentions through compensatory health beliefs was significant at low levels of health motivation (indirect effect=–0.0902, 95% CI –0.1985 to –0.0237), but not at high levels of health motivation (indirect effect=–0.0045, 95% CI –0.0797 to 0.0615; see Figure 5).

**Table 1:** Results of the moderated mediation analysis (not mean-centered).

Source			Coeff/Effect (SE)^a^	95% CI	*t* (*df*)	*P* value
**Outcome variables**						
	**Compensatory health beliefs**					
		Constant	3.30 (0.32)	2.67, 3.93	10.30 (393)	<.001
		Hedonic editing opportunity	1.11 (0.49)	0.14, 2.08	2.24 (393)	.03
		Health motivation	–0.04 (0.06)	–0.16, 0.08	–0.69 (393)	.49
		Hedonic editing opportunity × health motivation	–0.17 (0.09)	–0.35, 0.01	–1.82 (393)	.07
	**Health behavior intentions**					
		Constant	5.38 (0.37)	4.66, 6.10	14.66 (392)	<.001
		Compensatory health beliefs	–0.21 (0.05)	–0.31, –0.11	–4.04 (392)	<.001
		Hedonic editing opportunity	–0.28 (0.50)	–1.27, 0.71	–0.56 (392)	.58
		Health motivation	0.10 (0.06)	–0.02, 0.22	1.59 (392)	.11
		Hedonic editing opportunity × health motivation	0.02 (0.10)	–0.17, 0.21	0.22 (392)	.83
**Conditional indirect effect^b^**						
	**Value of the moderator**					
		Low health motivation (mean–1SD=3.94)	–0.09 (0.04)	–0.20, –0.02		
		Health motivation (mean=5.16)	–0.05 (0.03)	–0.12, –0.00		
		High health motivation (mean+1SD=6.38)	–0.00 (0.03)	–0.08, 0.06		
	**Index of moderated mediation**		0.04 (0.02)	0.00, 0.09		

^a^Coefficient for outcome variables and effect for conditional indirect effect.

^b^Of hedonic editing opportunity on health behavior intentions through compensatory health beliefs at values of the moderator health motivation.

## Discussion

### Principal Findings

Despite the growing popularity of digital self-tracking apps, to our knowledge this study is the first one exploring how spatial distance in the presentation of multiple self-tracking outcomes (physical activity and diet) affects users’ responses to their self-tracking outcomes. Building on principles of hedonic editing [23], this study has provided initial evidence that a spatially close presentation of mixed-gain self-tracking outcomes or a spatially distant presentation of mixed-loss self-tracking outcomes facilitate hedonic editing of one’s self-tracking outcomes. Highlighting potentially negative consequences of hedonic editing, this research has demonstrated that, particularly for users with low health motivation, the opportunity to hedonically edit one’s self-tracking outcomes reduces future health behavior intentions through the formation of compensatory health beliefs.

Specifically, we found that spatial distance affects users’ attention allocation and resulting memory of self-tracking outcomes consistent with principles of hedonic editing [23]. As such, in the case of a mixed-gain outcome profile, participants tended to systematically underestimate the small negative outcome when the outcomes were presented spatially close to versus distant from one another. In the case of a mixed-loss outcome profile, participants recalled the small positive outcome more accurately when the outcomes were presented spatially distant versus close to one another. This memory bias indicates that spatial distance between mixed self-tracking outcomes facilitates hedonic editing (ie, the mental integration of the small loss into the larger gain in the case of a mixed-gain outcome as well as the segregation of the small gain from the large loss in the case of a mixed-loss outcome and indicates that spatial distance facilitates to view one’s self-tracking outcomes in the most positive light). This finding is in line with previous research, which has proposed that attention allocation is a necessary condition of hedonic editing and has shown a systematic hedonic editing bias in users’ memory of temporally close or separate gains and losses in the context of gambling [26]. This study extends this previous work by demonstrating a hedonic editing bias for the memory of spatially close or separate gains and losses in the context of self-tracking, hence generalizing the finding to another type of experience and distance manipulation.

Moreover, this research offers a novel explanation of how a hedonic editing opportunity might affect users’ health-related behavioral intentions. Specifically, we provide initial evidence for a motivated cognitive justification mechanism and showed that particularly users’ with a lower health motivation form compensatory health beliefs in response to the opportunity to hedonically edit their outcome, hence leading to reduced health behavior intentions. Thus, for users with a lower health motivation, compensatory health beliefs help to legitimate the hedonic reflection of their self-tracking outcomes and accordingly to justify their reduced intentions for future health-related behaviors in response to their self-tracking outcomes. This finding supports the assumption that “there may be individual differences in the employment of hedonic editing strategies” ([26], p 82; see also [31]) and addresses the call for research to include measures of motivation in order to explain differences in the occurrence of hedonic editing and resulting justification processes of irresponsible behavior [26]. The finding that users’ levels of health motivation moderated the association between hedonic editing opportunity and compensatory health beliefs extends previous research that has provided evidence for the compensatory health beliefs model [32] in a dietary context and documented that autonomous weight-loss motivation decreases compensatory dietary beliefs [33].

This research adds to initial work exploring the effectiveness of self-tracking apps to encourage health behavior change [18] and contributes to literature on hedonic editing [23,24]. As such, this research transfers hedonic editing principles to a novel context, namely the context of self-tracking, and thus extends previous work that has investigated the use and consequences of hedonic editing principles in the context of price changes [34], emotionally impactful events [35], multiple time losses [36], or the evaluation of a gambling experience [26]. Moreover, this study extends previous research examining the influence of temporal distance on hedonic editing [26,27] by examining whether spatial distance can also facilitate the segregation or integration of outcomes as postulated by hedonic editing principles.

### Limitations and Future Research

The limitations of our study offer various avenues for future research. First, we used fictive self-tracking outcomes to create standardized experimental conditions. However, it would be interesting to know whether the observed effects also occur when users view their actual self-tracking outcomes. Second, our study focused on the immediate effects of spatial distance on hedonic editing biases and resulting health behavior intentions and thus cannot provide insights on longitudinal or actual behavioral effects. Accordingly, future research should investigate how spatial distance in the presentation of goal-related outcomes affects health behavior intentions over time and/or how it affects actual physical activity or dietary behavior. Third, we considered self-tracking outcomes from multiple domains that are measured along the same dimension (ie, calories). It would be interesting to examine if multiple self-tracking outcomes that are measured along different measurement units (eg, step count for physical activity and calories burned for dietary behavior) would yield similar hedonic editing effects. Fourth, the participants in our study were not made aware of the possibility that they may experience a hedonic editing bias. To provide insights regarding how the potential negative effect of hedonic editing opportunity on health behavior intentions might be mitigated, it would be worthwhile to investigate whether making users with low health motivation aware of their hedonic editing bias and the potential activation of compensatory health beliefs would attenuate the negative effect of hedonic editing opportunity on health behavior intentions. Fifth, with respect to the manipulation of spatial distance, this study only employed one potential way of presenting goal-related outcomes. In this regard, future research could investigate the integration/segregation of self-tracking outcomes in conditions where spatial distance is even greater (eg, using two separate domain-specific apps) or smaller (eg, one compound chart) than in this study. Finally, the characteristics of our sample limit the generalizability of our research’s findings. Our study employed a convenience online sample and shows how especially younger, majority female, normally weighted, and well-educated users respond to a close compared to distant presentation of mixed self-tracking outcomes and to the opportunity to hedonically edit one’s self-tracking outcomes. Accordingly, further studies might examine whether the observed effects change for older users or users who are less educated or heavily overweight.

### Implications

Considering that self-tracking apps constitute a promising, cost-effective tool to improve physical activity or dietary behaviors and to promote overall health outcomes [37], the findings of this research should be of interest for app designers and might be considered by health insurance providers, doctors, or even by app users. For example, designers of physical activity and diet tracking apps might consider developing an adaptive outcome presentation tool that aims to prevent hedonic editing biases by automatically maximizing the spatial distance between the presentation of both outcomes on the screen in the case of an overall mixed gain and by minimizing the spatial distance in case of an overall mixed loss. Spatial distance might be manipulated by displaying goal feedback horizontally compared to vertically or by presenting goal feedback separately in domain-specific halves of the screen or in two distinct user menu points compared to an integrative presentation. App designers might try to develop a tool that identifies users’ health motivations and accordingly adapt the app’s goal-related outcome presentation format for users with low health motivation. If the spatial distance in the app is difficult to adjust, app designers can think about integrating tools that assess users’ health motivation and that warns users with low health motivation about a possible hedonic editing bias when they encounter an outcome profile that would be conductive for such a bias.

### Conclusions

Our findings suggest that app design features such as spatial distance between the presentation of mixed positive and negative goal-related outcomes can provide users the opportunity to hedonically edit their self-tracking outcome profile (ie, to recall their self-tracking outcomes in a more positive light). As such, if users have a positive outcome in one domain and a smaller negative outcome in the other domain, a spatially close versus distant presentation of self-tracking outcomes could facilitate that users tend to recall the small negative outcome as being smaller than it actually was. Likewise, if users have a large negative outcome in one domain and a small positive outcome in the other domain, a spatially distant versus close presentation of self-tracking outcomes could facilitate that users recall the small positive outcome more accurately. Importantly, particularly among users with lower health motivation, the opportunity to hedonically edit one’s mixed self-tracking outcome profile leads to reduced health behavior intentions. Thus, to improve the effectiveness of self-tracking apps that employ goal-setting techniques for multiple domains, further studies are needed to determine the ideal distance between the presentation of mixed self-tracking outcomes on a digital device’s screen because it is conductive to prevent the occurrence of hedonic editing biases among users and to encourage health behavior intentions.

## Appendix

Multimedia Appendix 1 Details on measures and participant exclusion criteria.

## Abbreviations

BMI: body mass index
