# Supplementary material for: How Mobile App Design Impacts User Responses to Mixed Self-Tracking Outcomes: Randomized Online Experiment to Explore the Role of Spatial Distance for Hedonic Editing
Source: JMIR Mhealth Uhealth. 2018 Apr 11;6(4):e81. doi: 10.2196/mhealth.9055 (PMC5917081; doi:10.2196/mhealth.9055)
Supplement: Multimedia Appendix 1 [file mhealth_v6i4e81_app1.pdf]

Appendix A

Measures**:**

- **Health behavior intentions**: "In response to the results: How likely is it that you will have the following behavioral intentions" with four items on a visually anchored 7-point scale ( ) anchored left with "very unlikely" (= 1) and right with "very likely" (= 7): *I want to be more active in the near future*, *I want to do more sports in the near future*, *I want to eat healthier in the near future*, *I want to eat less high-caloric in the near future*; α = .70.
- **Health motivation**: "Please indicate your agreement to the following statements regarding the importance of activity and diet in your life": Four items on a visually anchored 7-point scale ( ) ranging from left 1 = *don't agree at all* to right 7 = *totally agree*: "Doing sports is an important element in my life", "I can easily live without doing sports" (reverse coded), "A healthy diet is important to me", "I eat unhealthy fast food very often" (reverse coded); reliability analysis revealed that the elimination of the last item increases α from .675 to .694. Thus, the last item was excluded.
- **Compensatory health beliefs**: "Please indicate your agreement to the following statements regarding activity- and diet-related behavior": Five items on a visually anchored 7-point scale ( ) ranging from left 1 = *don't agree at all* to right 7 = *totally agree*: "If I eat less or more healthily, it is not necessary to do a lot of sports", "It is OK to eat an unhealthy snack after having had a hard workout", "It is OK to eat more during lunch and dinner if one has skipped breakfast in the morning", "Skipping exercising in one week can be compensated for by exercising twice the next week", "Eating dessert is OK if one restrains eating during the main dish"; α = .68.
- **Manipulation check activity outcome**: "According to the presented outcome, what was your result in the domain of activity? I have…": One categorial item with three answer options: *…burned more calories than the set activity goal/…burned less calories than the set activity goal/…burned exactly as many calories as the set activity goal*.
- **Recall of activity outcome – Attention focus:** "How large was the absolute deviation (signs +/- not necessary) from the activity goal?" Open answer format: _____kcal.
- **Manipulation check diet outcome**: "According to the presented outcome, what was your result in the domain of diet? I have…": One categorial item with three answer options: …

consumed less calories than the set dietary goal/…consumed more calories than the set dietary goal/…consumed exactly as many calories as the set dietary goal.

- **Recall of diet outcome – Attention focus:** "How large was the absolute deviation (signs +/- not necessary) from the diet goal?" Open answer format: _____kcal.

Further Measures (not used in reported research)

- Outcome judgment: Two items on a visually anchored 7-point scale ( ) ranging from left = 1 to right = 7: *not satisfactory/satisfactory*, *unpleasant/pleasant*; *r* = .92.
- Outcome feeling: "When looking at the results, I feel…" with four items on a visually anchored 7-point scale ( ) ranging from left = 1 to right = 9: *unhappy/happy*, *dissatisfied/satisfied*, *uncomfortable/comfortable*, *displeased/pleased*; α = .96.
- Health behavior decision: "Which behavioral intention would be more likely in response to your results? I would rather…": One item on a visually anchored 7-point scale scale ( ) ranging from left = 1 to right = 7: *…focus on sports and activity/focus on a healthy diet*.
- Judgment focus: "Was your judgment of the results rather based upon the singular outcomes in the domain of activity and diet or on the overall result of the energy balance?": One item on a visually anchored 7-point scale scale ( ) ranging from left = 1 to right = 7: *overall energy balance/activity and diet*.
- App focus: "Imagine a friend would ask you about the app's function: According to your own perception, what would you tell her for which function the app is primarily good for?" One item on a visually anchored 7-point scale scale ( ) ranging from left = 1 to right = 7: *better control of activity and diet behavior/better control of health-related behavior*.
- App design: "This question concerns your vision for the app design: Would it be more comfortable for you to access the self-tracking scores in the domain of activity and diet either separately in a detailed views or together in an overview?" One item on a visually anchored 7-point scale scale ( ) ranging from left = 1 to right = 7: *separately (detailed view)/together (overview)*.
- Current health-related lifestyle: "How would you rate your lifestyle overall? Please indicate your agreement to the following statements regarding your overall lifestyle as it relates to health": Two items on a visually anchored 7-point scale ( )

ranging from left 1 = *don't agree at all* to right 7 = *totally agree*: "I consider my lifestyle to be very healthy" and "I always pay attention to a sufficient level of exercise and a healthy diet"; $r = .70$.

Manipulation check procedure and criteria for participant exclusion

We excluded a) participants who reported an extreme high or unreasonable low deviation from the determined caloric goals in the domain of activity or diet or b) participants who indicated a wrong outcome profile.

a) In particular, we excluded participants who reported to remember a calorie performance that was either extremely above or unreasonable below the actual outcome displayed in the outcome profile in the domain of activity or diet. To do so, we conducted boxplot analyses across the whole sample to identify extreme outliers in the distribution of participants' freely recalled goal deviations (kcal values) in the domain of physical activity and diet. Accordingly, based on the distribution of recalled values across all conditions and participants, participants who reported to remember an extreme deviation from the caloric goal in the domain of activity (reported goal deviation in kcal values ≥ 450) and/or diet (reported goal deviation in kcal values ≥ 1000) were identified and excluded. Regarding the lower bound of the box plots, no outliers were identified. Nevertheless, it is plausible to assume that participants who reported an extremely small deviation from the caloric goal in the domain of activity and/or diet (remembered kcal values ≤ 10) indicated such small kcal values due to experimental fatigue. That is, participants who reported that their goal deviation in the domain of physical activity and/or diet was smaller or equal ten calories might have just quickly typed in a numeric value to speed up finishing the experiment. Alternatively, participants who reported that their goal deviation in the domain of physical activity and/or diet was smaller or equal ten calories might just not have paid enough attention to the actual goal deviation as it was presented in the outcome profile.

b) A wrong outcome profile is indicated when participants cannot correctly specify which outcome profile they had actually seen during the experiment. To do so, we included categorial manipulation check items. These items asked participants to indicate for each of the two domains (i.e., physical activity, diet) whether according to the presented outcome profile their result was a gain (i.e., *burned more calories than the set activity goal*, *consumed less calories than the set dietary goal*), a loss (i.e., *burned less calories than the set activity goal*, *consumed more calories than the set dietary goal*) or whether they had exactly matched the goal (i.e., *burned exactly as many calories as the set activity goal*, *consumed exactly as many calories as the set dietary goal*). This procedure helped to ensure that we only included those participants in our analyses where the experimental manipulation was effective, i.e., those participants, who paid sufficient attention to the experimental manipulation and who

consequently could correctly indicate whether they had a gain or a loss outcome in the domain of physical activity and, respectively, diet.

For example, in the mixed-gain conditions, the presented outcome profile showed participants that they burned less than their activity goal (loss) and that they consumed less than their dietary goal (gain). Accordingly, participants indicated a wrong outcome profile if they specified that - according to the presented outcome profile - they had "*burned more calories than the set activity goal*" or that they had "*burned exactly as many calories as the set activity goal*". Moreover, participants indicated a wrong outcome profile if they responded that they "*consumed more calories than the set dietary goal*" or that they "*consumed exactly as many calories as the set dietary goal*". As such, participants in the mixed-gain conditions only indicated a correct outcome profile if they specified that according to the presented outcome profile they had "*burned less calories than the set activity goal*" on the manipulation check item regarding the activity outcome as well as if they picked the response option "*consumed less calories than the set dietary goal*" on the manipulation check item regarding the diet outcome. Similarly, in the mixed-loss conditions, a correct outcome profile was indicated only if participants stated that according to the presented outcome profile, they had "*burned more calories than the set activity goal*" as well as that they had "*consumed more calories than the set dietary goal*". Accordingly, an incorrect outcome profile was indicated when participants picked any of the other two answer options for their result in the domain of activity and diet.
